# Infant Exposure to Metals through Consumption of Formula Feeding in Mekelle, Ethiopia

**DOI:** 10.1155/2018/2985698

**Published:** 2018-04-24

**Authors:** Tadele Eticha, Melat Afrasa, Getu Kahsay, Hailekiros Gebretsadik

**Affiliations:** School of Pharmacy, College of Health Sciences, Mekelle University, Mekelle, Ethiopia

## Abstract

This study aimed at determination of heavy metals (cadmium, lead, and zinc) in milk-based infant formulas collected from Mekelle, Ethiopia, and their associated health risks to the infants through consumption of these products. The infant feeding samples were dry-ashed in a muffle furnace followed by digestion in nitric acid and the resulting solutions were analyzed by flame atomic absorption spectrophotometer. Cadmium was not detected in the samples while the levels of lead and zinc ranged from not detected value to 0.103 mg/kg and from 27.888 to 71.553 mg/kg, respectively. The estimated daily intake values and the health risk indices of both metals were below their respective safety limits and the threshold of 1, respectively. These findings show low infant health risk of these metals through consumption of these products. Nevertheless, regular monitoring of infant formula for toxic metals is required since infants are potentially more susceptible to metals.

## 1. Introduction

Infant feeding deserves top priority in child healthcare and appropriate infant formula can prevent millions of deaths occurring from infantile gastroenteritis and malnutrition [1]. Infants are potentially more sensitive to environmental contaminants such as toxic heavy metals because of their significant higher absorption of contaminants than adults and a lower threshold for adverse effects. The developing systems, including nervous system development, of babies are vulnerable to toxic heavy metals particularly lead [2, 3]. Even at low concentrations of exposure heavy metals bioaccumulate in vital organs and persist in adulthood [3–5]. The impact of lead exposure at low levels has been entrenched and concentrations below toxic have exhibited impairment of neurobehavioral and learning process in infants [3]. Exposure to cadmium has a great contribution to kidney toxicity, cancer, renal stone formation, neurological effects, disturbances in calcium metabolism, and diseases [6–8]. Even though zinc is essential micronutrient and has different biochemical functions in living organisms; this metal can be dangerous when taken in excess [9].

The presence of cadmium, lead, and zinc in infant formula products has been reported in the literature. A wide concentration range of cadmium and lead from undetectable level to 0.4 mg/kg and 0.142 mg/kg in infant feeding has been recorded in different countries, respectively [10–16]. However, higher levels of both metals have been investigated in cereal-based, milk-based, and mixed baby feeding products in Turkey [17]. The concentrations of zinc have been reported from 0.92 to 52.3 mg/kg in similar products in the literature [10, 11, 16, 18]. Such findings suggest strong regular monitoring of infant formulas for toxic heavy metals. The manufacturers of the products should also be conscious of the toxicological effects of heavy metals and greater absorption of these metals in children to prevent potential contaminants and protect the users. The assessment of dietary exposure to contaminants in baby foods is required to ensure healthy infant growth [19].

The Joint FAO/WHO Expert Committee on Food Additives (JECFA) has set the provisional tolerable weekly intakes (PTWI) for cadmium and lead at 7 and 25 *μ*g/kg bw/week, respectively [20]. The Scientific Committee on Food (SCF) extrapolated tolerable upper limit of 7 mg/day of zinc for children aged 1–3 years from adults' tolerable upper limit because of unestablished zinc limit for them [21].

The purpose of this study was to investigate the levels of metals such as cadmium, lead, and zinc in various brands of infant formulas in Mekelle, Ethiopia, and their associated health risks in infants through taking these products.

## 2. Materials and Methods

Nitric acid (Sigma-Aldrich, Germany) and analytical grade stock standards of elements (Inorganic Ventures, USA) were used in this work. Maple furnace (Carbolite Gero, UK) and hot plate (Edu-Lab, UK) were employed.

### 2.1. Sample Collection

Five different brands of dry powdered infant formulas were collected from supermarkets in Mekelle, Ethiopia, between March and April 2017. The infant formulas were the most widely available in the market, which are intended for consumption from six months until one year. All products were milk-based types and packed in metal containers. Samples were coded for ease of identification.

### 2.2. Sample Digestion and Analysis

All samples were digested in duplicate following the procedures described in the literature [22]. Five grams from each milk-based sample was placed in different crucibles and heated in a maple furnace at 550°C for 3 hours to vaporize all other constituents and leave the heavy metals as a pure ash. The ash was cooled to room temperature before being dissolved in a 5 ml solution of nitric acid (70%) to compound with the heavy metals, if present. The solution was subsequently heated and evaporated to half its volume using a hot plate. The resulting solution was then poured into a volumetric flask and topped up to 25 ml with distilled deionized water. The digested samples were analyzed using flame atomic absorption spectrophotometer (AA240FS, Varian, Australia).

### 2.3. Quality Control

Sample containers and all glassware were soaked in 10% nitric acid for 24 hours and then rinsed with distilled deionized water thoroughly. Analytical grade reagents were used throughout the experiment. Standard solutions were prepared from stock standard 1000 mg/L. The calibration curves were constructed from five points (*r*^2^ = 0.998 to 0.9996). A matrix spike recovery was performed by adding standard solutions of metals to known amount of sample. The spiked samples were digested and then analyzed (percentage recoveries = 95.5% to 104.6%). Limit of detection (LOD) in this study was determined as three times the pooled standard deviation of six runs of blank measurements. LODs for Cd, Pb, and Zn were 0.0096, 0.008, and 0.0081 mg/kg, respectively. Blank samples were also prepared using the same procedures as those for the samples.

### 2.4. Daily Intake of Metals and Health Index Calculation

Estimated daily intakes of metals were calculated using the concentrations of metals obtained from the samples, daily intake of infant formulas, and average body weight. The average daily consumed powder infant formula from feeding tables and dosages recommended by manufacturers at 6 and 7–12 months were 137 and 108 g/day while the mean body weights of infants in the literature at both ages were 7.5 kg and 10 kg, respectively [17]. The health risk indices were estimated by dividing mean daily intake values of heavy metals by their safe limits to assess risk exposure to these metals in the baby formula.

### 2.5. Statistical Analysis

The data were analyzed using one-way analysis of variance (ANOVA) and Tukey post hoc test at 95% confidence level. SPSS statistical package version 20.0 was used for statistical analysis.

## 3. Results and Discussion

### 3.1. Levels of Metals in Infant Formula

The levels of elements in five different milk-based infant formulas, targeted for infants aged 6 months to 1 year, are presented in Table 1. Although cadmium was not detected in all brands of infant formulas in this work which is in agreement with the findings from the surveys done in Addis Ababa, Ethiopia [10], and Kenya [15], other studies in different origins investigated the presence of cadmium in formula feeding [13, 14, 16, 17] as shown in Table 2. The cadmium levels recorded in infant formula products collected from the Nigerian market were 0.05–0.4 mg/kg [11].

Table 2 illustrates a comparison of metals concentrations in infant formulas investigated in this work with reported data in the literature. The levels of lead in the samples in the present survey ranged from being not detected to 0.103 mg/kg, which is consistent with the results obtained from other studies [12–16]. Aguzue et al. [11] reported 0.08–0.23 mg/kg of lead levels in infant formula in Abuja, Nigeria, whereas lower amounts, 0.00375–0.0249 mg/kg, of lead were recorded in similar samples in Turkey [17]. However, the study conducted in Addis Ababa, Ethiopia, revealed that the levels of lead in the powdered infant formulas were below the detection limit [10].

The contents of zinc in the infant formula in this work were within the range of 27.888 to 71.553 mg/kg, which is similar to the findings of another study [16]. However, lower levels of this metal were found in baby formulas in various studies done in Addis Ababa, Ethiopia [10], Nigeria [11], and Turkey [18]. The variation of the metals in infant feeding could be related to the types and origins of raw materials, manufacturing processes, and adulteration of the products [23].

One-way analysis of variance revealed that there were significant differences between various brands of infant formula products for their lead and zinc contents (*p* < 0.05). The mean levels of measured lead and zinc were significantly higher in products B and L, respectively, while infant formula P had significantly lower concentration of zinc content. Navarro-Blasco and Alvarez-Galindo [24] reported similar results that there were significant variations of lead concentrations across the infant formula brands. This difference could be attributed to the variation of raw materials used in manufacturing, production practices, finished products, and packaging containers used by infant formula manufacturers [25].

### 3.2. Risk Exposure Assessment

Table 3 presents the EDI and HI values of both lead and zinc contents in the samples. The estimated daily intake values of lead found in the baby formula ranged from 0.292 to 1.881 *μ*g/kg bw/day and 0.173 to 1.112 *μ*g/kg bw/day for infants during the formula feeding ages of 6 and 7–10 months, respectively. The provisional tolerable weekly intake (PTWI) of lead set by the Joint FAO/WHO Expert Committee on Food Additives is 25 *μ*g/kg bw/week, and hence its tolerable daily intake (TDI) value is 3.6 *μ*g/kg bw/day [20]. The EDI values for lead were below the PTWI and/or TDI established by JECFA. Similar findings were investigated from the most consumed infant formulas in Europe, where the average PTWI for lead was 2.6 and 5.8 *μ*g/kg bw/week for infants during the formula feeding (0–4 months) and weaning period (5–9 months), respectively [16]. Mehrnia and Bashti [14] also reported the mean estimated daily intake of 0.37 *μ*g/kg bw/day for lead in infant foods commercially available in Iran. The contributions of lead to PTWI from different types of infant formulas in the range of 16.6%–29.3% were investigated in Spanish [26]. Other studies done in different origins recorded higher concentrations of lead in infant formula [27–29]. The adverse effect of lead exposure at low levels has been well established and concentrations below toxic level have been shown to contribute to behavioral and learning issues [3]. Hence, monitoring of this metal in infant formula is critical.

The EDI values of zinc obtained from the samples in the present work were in the range of 0.509 to 1.307 mg/kg bw/day and 0.301 to 0.773 mg/kg bw/day for infants aged 6 and 7–10 months, respectively. There is no established tolerable upper limit for zinc in infants. However, the Scientific Committee on Food estimated 7 mg/day of tolerable upper limit for zinc from that of adults to 1–3-year-old children [21]. Overall, the calculated values for this metal were below the limit. Higher mean daily intake, 4.5 mg/day, of zinc was recorded in infant formula samples in Europe [16].

## 4. Conclusion 

The findings of this study investigated the presence of lead and zinc in the infant formulas and their estimated daily intakes were less than their respective safety limits. Furthermore, the health risk indices of both metals were below the threshold of 1 at mean exposure, which implies low health risks of these metals to the general infants upon consumption of the formula feeding. However, regular assessment of infant formula products for toxic heavy metals is essential as infants are more sensitive population.

## Figures and Tables

**Table 1:** Concentrations of metals (mg/Kg) in infant formula (mean ± SD).

Infant formula code	Cd	Pb	Zn
Infant formula B	ND^*∗*^	0.103 ± 0.091	45.732 ± 0.555
Infant formula L	ND	0.051 ± 0.058	71.553 ± 1.104
Infant formula M	ND	0.016 ± 0.027	39.873 ± 0.921
Infant formula N	ND	0.063 ± 0.065	42.127 ± 1.014
Infant formula P	ND	ND	27.888 ± 0.542

^*∗*^ND: not detected.

**Table 2:** A comparison of metals levels (mg/kg) in infant formulas with reported data in the literature.

Origin/market site	Cd	Pb	Zn	Reference
Ethiopia	ND	ND–0.103	27.888–71.553	This study
Ethiopia	ND	ND	32.53–42.98	[10]
Nigeria	0.05–0.4	0.08–0.23	6.82–17.19	[11]
Nigeria	–	ND–0.142	–	[12]
Pakistan	0.0042–0.0123	0.0287–0.097	–	[13]
Iran	0.0403–0.058	0.03183–0.03185	–	[14]
Kenya	ND	0.018–0.059	–	[15]
EU market	0.0033–0.0045	0.0082–0.0439	36.2–52.3	[16]
Turkey	ND-0.00749	0.00375–0.0249	–	[17]
Turkey	–	–	0.92–37.2	[18]

**Table 3:** The estimated daily intake (EDI) values of metals from commercial infant formulas and the health risk index (HI).

Age in months	Metal	Safety limit	Infant formula code, EDI					Mean EDI	HI
			B	L	M	N	P		
6	Pb^*∗*^	3.6	1.881	0.932	0.292	1.151	-	1.064	0.30
	Zn^*∗∗*^	7	0.835	1.307	0.728	0.769	0.509	0.83	0.12
7–12	Pb	3.6	1.112	0.551	0.173	0.68	-	0.629	0.17
	Zn	7	0.494	0.773	0.431	0.455	0.301	0.614	0.09

^*∗*^Lead level in *μ*g/kg bw/day. ^*∗∗*^Zinc level in mg/kg bw/day.

## Data Availability

The data used to support the findings of this study are available from the corresponding author upon request.
